# The Universal Homœopathic Annual of 1894

**Published:** 1895-11

**Authors:** 


### BOOK NOTICES.

THE UNIVERSAL HOMŒOPATHIC ANNUAL OF 1894. Edited by François Cartier, M. D., Paris, France, with the collaboration of several associate editors, assisted by physicians specially attending to the translating department of German, French, Spanish, Italian, Russian, Dutch, and Danish. Issue of 1895. *Éd. Crêté Imprimerie Typographique Corbeils* (*Seine-et-Oise*). Price, $3.00. Address Dr. F. Cartier, 18 Rue Vignon, Paris, France.

We owe an apology alike to the editor and to the profession as well for not previously noticing this book. The pressure of other work and the need of a careful examination of the book itself must be our excuse.

As the title-page shows, it is a collection of all the best points of information upon homœopathic practice culled from the homœopathic journals of the world. Dr. Carter was assisted in this immense task by a select body of associates, who are as follows: Dr. Timothy Field Allen, Giuseppe Bonino, George Burford, Henry C. Houghton, Sutcliffe Hurndall, Horace F. Ivins, Pierre Jousset, John R. Kippax, W. B. Van Lennep, A. B. Norton, V. L. Simon, Selden H. Talcott, Alphonse Teste, Alexander Villers.

Their combined efforts have been to condense all the practical things of all the homœopathic journals into a book of 500 pages. It may be well to state that, though prepared in France by Frenchmen, it is in the English language. We regard it as a most valuable addition to homœopathic literature.

Looking over its pages, we notice a number of valuable points. A few of these we herewith detail. On page 41 there is a discussion as to the chemical nature of homœopathic Causticum. It will be remembered that Hahnemann, in the *Chronic Diseases*, directs that it be prepared by distilling a solution of Bisulphate of Potash with slaked lime. It has always been a matter of doubt what is the nature of the distillate to which Hahnemann gave the name of Causticum. According to the *Annual*, it is a weak solution of Potash.

The book has two main departments, one of remedies in alphabetical order and another of diseases.

In the second department, under Angina-pectoris, we pick out the following item: Tabacum and Spigelia are the two principal remedies for angina. "Tobacco produces symptoms which are altogether similar to those of angina-pectoris." Then reference is made to the history of the vessel which was returning from Havana with a cargo of tobacco, when nearly all the crew were taken with symptoms of angina-pectoris. Several cases of cure of angina with Tabacum in the third trituration are mentioned, and the assertion made that "Spigelia presents a complete picture of the symptoms of angina."

Angina is always connected with arterio-sclerosis. Professor Lancereaux claims that alcoholism is never the cause of arterio-sclerosis, which is, instead, due to herpetism, or a disposition to herpes, equivalent to Hahnemann's psora. "My master, Jean Paul Tessier, Sr., used to say," remarks Pierre Jousset, "that arterio-sclerosis was always a gouty affection, and I find this expression more medical than herpetism, but he also knew that alcoholism is one of the most powerful occasional causes to localize the gout (we will say herpetism if Dr. Lancereaux wishes) on the arteries."

The value of Lycopus-virgicus in cardiac asthma is shown in one case where the patient could not lie down for two days. Her chest was bared, and her hands clenched into the integument trying to assist the muscles of respiration. Lycopus relieved.

A case of dropsy is related where Apocynum-cannabinum entirely cured. Two old men similarly affected were successfully treated with Apocynum.

Moschus is the great fainting remedy. The patient faints from the slightest causes. She has globus hystericus, like Ignatia, palpitation of the heart, constriction of the chest, spasmodic suffocating spells, coldness of the skin, nervous trembling, and aggravation after sleep.

Erigeron is regarded as almost a specific in hemorrhage. No reliable indications can be given, as none are known. Two cases only are known where it failed, one being caused by uterine polypus, and the other by cancer.

"That tired feeling" is the subject of some remarks by Dr. Frank Kraft, well known as the editor of *The American Homœopathist*. He gives indications for several remedies. They are very clear and soundly homœopathic. It is a most excellent article.

Chapter fourth relates to the treatment of animals by Homœopathy.

Carbolic-acid poisoning is noticed and mention made of Dr. Edmund Carleton's paper on that subject published in THE HOMŒOPATHIC PHYSICIAN for March, 1894, page 77. Dr. Carleton's assertion that the antidote is Cider-vinegar is confirmed, but Magnesia-sulph. is considered even better than Vinegar.

Scopolamine is mentioned as better than Atropine for dilating the pupil, and the reasons are given in detail.

Nasal mucus is claimed to be a powerful destroyer of the germs in the air. That it not only filters them out of the air entering the lungs, but it destroys them also.

Much more of an interesting character might be extracted from this book for the benefit of our readers, did space permit. The foregoing items, however, show the scope of the book and its value. We regret that it has not a more perfect index. The absence of an index always handicaps the most valuable book.

